# Trimodally treatment for stage IIIa NSCLC patients increases survival while not effecting surgical mortality or complexity

**DOI:** 10.1186/s13019-018-0829-z

**Published:** 2019-01-09

**Authors:** Aravot Dan, Barac D. Yaron, Krutzwald-Josefson Efrat, M. Allen Aaron, Flex Dov, Peled Nir, R. Kramer Mordechai, Peysakhovich Yuri, Saute Milton

**Affiliations:** 1 Department of Cardiothoracic Surgery, Rabin Medical Center, Beilinson Campus, Petach-Tikva, Israel; 2 Department of Oncology, Rabin Medical Center, Beilinson Campus, Petach-Tikva, Israel; 3 Department of Pulmonary Medicine, Rabin Medical Center, Beilinson Campus, Petach-Tikva, Israel; 4 Sackler Faculty of Medicine, Tel Aviv University, Tel Aviv, Israel

**Keywords:** Pneumonectomy, Neo adjuvant therapy, Chemo-radiation

## Abstract

**Introduction:**

Advanced non-small cell lung cancer (NSCLC) is still a therapeutic challenge as the 5-year survival is under 30%. The optimal treatment regimen is still under debate.

**Hypothesis:**

Neo adjuvant (NA) treatment given pre-pneumonectomy does not increase surgical complexity or peri-OP mortality while it has a potential to increase long term survival.

**Methods:**

We have conducted a retrospective study of 169 patients who underwent a pneumonectomy for NSCLC between January 2005 to December 2015 and focused on stage IIIa patients; a cohort of 51 patients, 30 which received neo adjuvant chemo-radiation (NA group) prior to pneumonectomy and 21 patients who had undergone pneumonectomy followed by adjuvant treatment (Adjuvant group). Surgical complexity and short- and long-term survival were evaluated. Surgical complexity was assessed by surrogates as surgery duration, hospitalization length and interdepartmental transfer.

**Results:**

While no statistically significant differences were found in surgery duration, hospitalization length, morbidity in the 1st year post-OP and the peri-OP mortality; The long term beneficiary effect among the neo adjuvant patients was clear; while 30% of the NA patients were alive 8 years post-OP, there were no survivors in the adjuvant group 5.5 years post-OP.

**Conclusion:**

We conclude that while NA treatment has no effect on operation complexity, peri-OP mortality or post-OP morbidity; its impact on long term survival is protuberant, therefore, we believe that NA treatment should be considered as the treatment of choice in advanced NSCLC in need for pneumonectomy.

## Introduction

Lung cancer is the leading cause of cancer-associated deaths in the USA [1]. As the course of this disease is rapid, mortality rates are almost identical to those of incidence, moreover, in 25% of the patients disease diagnosis is made in advanced stages, e.g. stage III and up [2]. A specific group of patients is challenging the physicians; the IIIa group, where patients can range from T1–4 and N1-N2 mixtures of disease, thus choosing the ideal protocol of treatment is an ambitious task. The surgical treatment for lung cancer ranges from segmental resection, lobectomy and up to pneumonectomy, where the latter represents less than 15% of all lung cancer associated surgeries [3]. While nowadays pneumonectomy is considered a safe procedure, this was not the case in the past where attempts to perform pneumonectomy were complicated due to hemorrhage, sepsis and lack of a durable bronchial closure[4]. Nonetheless, the use of pneumonectomy in the treatment of non-small cell lung cancer (NSCLC) is still controversial. Surgery as a treatment modality is being assisted by both chemotherapy and radiation, which serve as adjuncts; while the combination between the three has a potential for better patient prognosis, the way this combination should work is still debatable. At present, pneumonectomy is indicated for fewer patients, as smaller resection demonstrated similar survival with less associated morbidity. Literature review reveals that the associated mortality rate is between 8 and 15% and that complication rate spans between 17 and 47% [5]. Furthermore, when added to chemotherapy and radiation it’s advantages are in doubt and no consensus exists [6]. Even when the tri-modality is investigated the results are conflicting; while some point on survival benefit other only demonstrate advantage in disease progression free survival [7].

As adding the neoadjuvant chemo-radiation treatment to the equation only increases the uncertainty in relation to the complication and mortality rate, this retrospective study investigated the morbidity, mortality and survival benefit of patients diagnosed with stage IIIa of NSCLC and treated with pneumonectomy with or without neoadjuvant treatment at our center.

Our aims were:

1. Demonstrate that neoadjuvant treatment before pneumonectomy does not increase the surgical mortality or complexity.
2. Assess the long-term morbidity and survival benefit following neoadjuvant treatment + pneumonectomy Vs pneumonectomy + adjuvant treatment in stage IIIa NSCLC patients.

## Methods

### Patients

The clinical records of 169 patients who underwent a pneumonectomy from January 2005 to December 2015 our center, were retrospectively reviewed following an institutional Helsinki committee approval.

### Inclusion criteria

Patients were included in the study if they had undergone a complete resection (R0) and there were precise data about the pretreatment tumor and patient characteristics and comorbidities, a detailed post-surgery pathological report and precise data about postoperative complications, neoadjuvant or adjuvant therapy and treatment outcome.

### Preoperative work-up

The preoperative work-up for the assessment of the local extent of the lung cancer was the same in all patients (standard clinical and laboratory investigations, bronchoscopy, high-resolution computed tomography scan of the thorax, upper abdomen and brain, cardiac echography and respiratory function tests).

For the staging of the mediastinum and a thorough search for distant metastases, a positron emission tomography (PET) scan was performed for all patients. A mediastinoscopy was performed only for PET-positive patients or for patients who had lymph nodes > 1 cm. For patients with moderate and severe chronic obstructive pulmonary disease (COPD), we calculated the predicted postoperative forced expiratory volume in 1 s (ppoFEV1) using a perfusion lung scintigraphy with quantification of perfusion for each lung. A value of ppoFEV1 > 40% was accepted as the lower limit for safe lung resection. For all patients with ppoFEV1 > 40%, a peak oxygen consumption of 15 ml\kg- 1\min^− 1^, served as a cut-off value for safe resection, according to the current guidelines. The tumors were classified and staged according to the 2009 revision of the International System for Staging of Lung Cancer.

### Operation

All pneumonectomies were performed via posterolateral thoracotomy. The bronchial closure was performed using a stapler; all stumps were covered with a pediculate pleural flap. After undergoing pneumonectomy, all patients were extubated in the operating theatre and transferred to a post-anesthesia or intensive care unit. When the postoperative course was uneventful, patients stayed in the surgical ward for up to 8 days.

### The Neoadjuvant protocol

1. The chemo-radiation protocol was based on the SWOG regimen of Gandara et al. (JCO 96′) and consisted of Cisplatin (50 mg/m^2^ - at days: 1,8,29,36) and Etoposide (50 mg/m^2^ – at days: 1–5,29–33. This was given with concurrent radiotherapy to 72Gy in 2 Gy daily fractions. Radiotherapy was delivered with IMRT (intensity modulated radiotherapy).
2. Restaging FDG-PET/CT scan was preformed 4–6 weeks following concurrent CRT to determine the suitability of the patients for surgery. Once the mediastinum was free of active disease, and no new disease in any other new site, meaning that the patient stage was downgraded, patients were referred for surgical resection.

### The adjuvant protocols

In the adjuvant group the patients that were stage II and up received the following adjunct chemotherapy protocol: Cisplatin (75 mg/m^2^ on day 1 every 3w) and Vinorelbine (25 mg/m^2^ on day 1and 8 every 3w). If cisplatin was contraindicated the patients got instead carboplatin (AUC 5.5 on day 1 every 3w).

### Postoperative course

Non-lethal postoperative complications occurring during the first month (30-day morbidity), postoperative deaths occurring during the first month (30-day mortality) as well as survival years after, were recorded. Complications consisted of sub-cutaneous emphysema, bronchopleural fistula, pneumonia, respiratory failure, acute respiratory distress syndrome, pulmonary emboli, myocardial infarction, cardiac arrhythmia, stroke, recurrent nerve palsy, hemorrhage, chylothorax, parietal infection and miscellaneous. Follow up was completed for all patients on December 31st 2015.

### Statistical analysis

The statistical analysis was generated using SAS Software, Version 9.4. Continuous variables are presented using Mean ± Std. Categorical variables are presented as (N, %). Normality of continuous study variables was assessed graphically. For normal variables, T test (for two groups) or ANOVA (for more than two groups) were used to compare the value of the variables between study groups; for highly skewed variables, the Wilcoxon’s non-parametric test was used. Logistic regression was used to compare the value of categorical variables between study groups. Overall survival was assessed by Kaplan-Meier survival analysis, with the log-rank test. *P* < 0.05 was considered an indication of a statistically significant result.

## Results

### Study group

In total, a single surgeon (M.S) performed 169 pneumonectomies’ during the study period, 30 of which were accompanied by neoadjuvant chemo-radiation therapy (NA), while 139 were accompanied by adjuvant chemotherapy. We chose to focus on stage IIIa patients; the NA group patients were all clinically down-staged prior to the operation, the stage IIIa adjuvant group consisted of 21 patients (Got no treatment prior to the pneumectomy and thus were not downgraded before surgery). No significant inter-cohort differences were noted in patient demographics and baseline characteristics. The cohorts were almost equally distributed between genders (51.43 and 48.57% men in the Neoadjuvant and adjuvant cohorts, respectively), with baseline forced expiratory volume in 1 s (FEV1) of 69.39 and 72.88%, respectively (Table 1).

**Table 1:** Patient Characteristics

		N.A	Adjuvant	p
N P Stage IIIa		30	21	
Age	Mean	59.83	60.10	0.939
Male	N	18	17	0.220
	%	51.43	48.57	
FEV1(%)	Mean	69.39	72.88	0.644
Laterality of procedure				
Right		13	14	0.154
Left		17	7	
Histological type				
Adenocarcinoma		35.5	28.6	
Squamous cell carcinoma		48.4	28.6	
Metastatic thymoma		6.5		
Large cell carcinoma		9.6	9.5	
Malignant mesothelioma			28.6	
Bronchiolo-alveolar carcinoma			4.7	

### Operation complexity from a surgical point of view

**Fig. 1:**
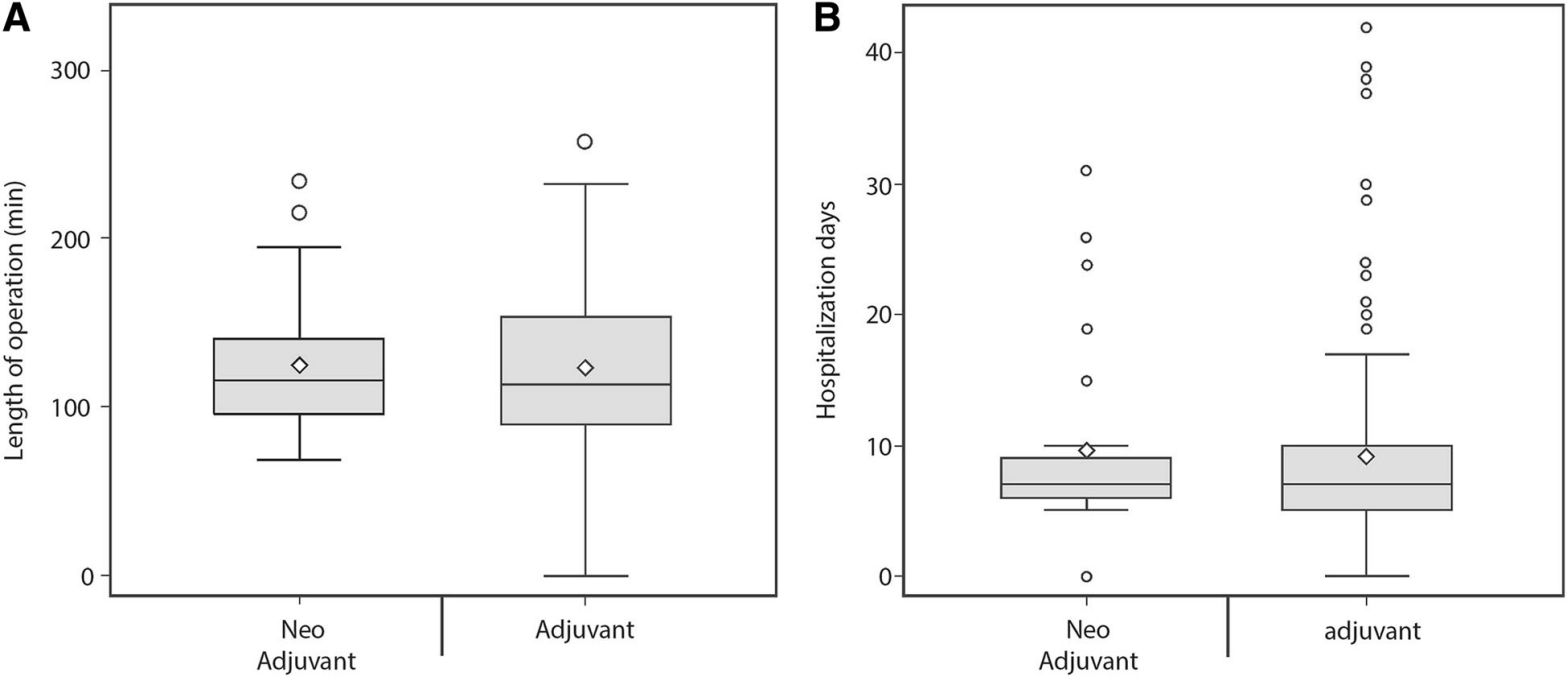
Operation complexity. Boxplot of the length of operation (**a**) and the length of hospitalization (**b**) post pneumonectomy. No significant difference was found between the study groups

Neoadjuvant treatment has been claimed to increase the complexity of the subsequent lung operation, thus, to address this we have recorded pneumonectomy operation length, from skin incision to skin closure (Open approach), as well as hospitalization length, as surrogate markers for operation complexity. No differences in surgery duration (~ 120 min) was noted between the two cohorts (Fig. 1a), suggesting no difference in operation complexity. Moreover, hospital length of stay was quite similar between treatment cohorts, averaging up to 8 days (Fig. 1b).

### Re-admission and transfer between departments in subsequent hospitalizations

**Fig. 2:**
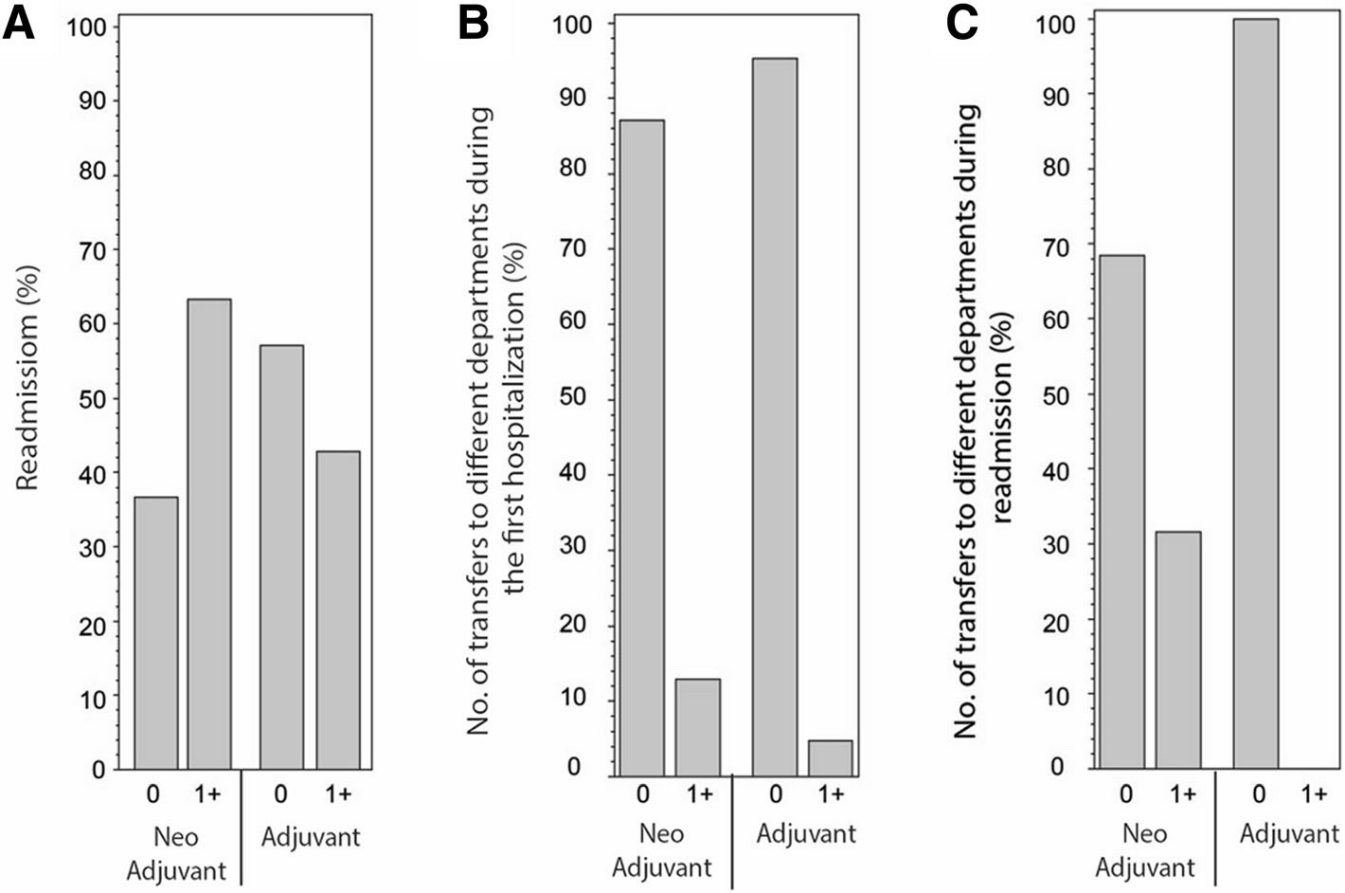
Cohort morbidity. **a** Patient readmission in the first-year post pneumonectomy. **b** Number of transfers to different departments during the first hospitalization (**c**) and in the readmissions to follow. 0 = No readmission/No transfer, 1+ = One or more readmission/Transfer. No significant difference was observed between the groups

Postoperative morbidity was monitored by readmissions as well as inter-hospital transfers between departments. We followed our patients for 365 days after surgery for hospital readmissions (Fig. 2a). The majority of the adjuvant patients (57.14%) did not require re-hospitalization, in contrast, 63.3% of the Neoadjuvant patients did require rehospitalization, (no significant difference). When comparing the number of interdepartmental transfers, the vast majority of patients in both cohorts remained at the same department during the initial postoperative period, thus pointing out a reduced number of immediate post-op complications needed to be addressed by other disciplines (Fig. 2b).

In contrast, the number of interdepartmental transfers during subsequent hospitalizations was 0 in the adjuvant group and was 31.58% for the Neoadjuvant group (*p* = 0.07) (Fig. 2c).

### Short- and long-term survival

**Fig. 3:**
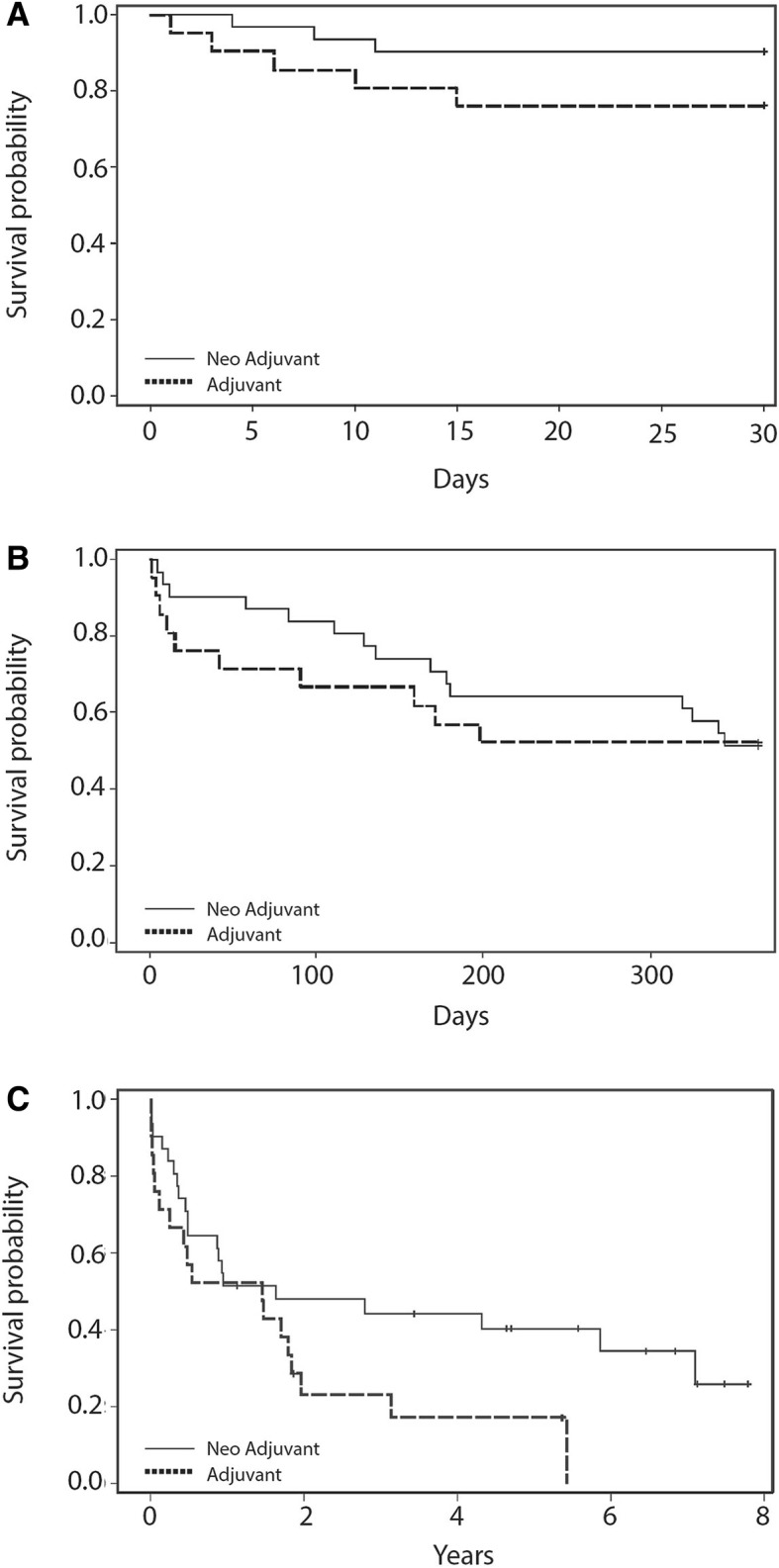
Cohort survival. Kaplan Meier curves for survival segregated by the treatment group; **a**. 30 days. **b**. 1-year **c**. 8 years. A significant difference in survival was found 8 years post-OP, *p* = 0.0343

Upon examination of the postoperative course, a high 30-day survival rate was observed, importantly, no difference was observed between the groups in regards to peri-OP mortality. Survival rates gradually declined over the first year following surgery, at an equal pace for both cohorts, reaching a mean survival rate of roughly 60% for both cohorts, by the end of the 1st year (Fig. 3a, b). In contrary, at 8-year post-OP the survival rate was significantly different between the cohorts; while 30% of the neoadjuvant patients were still alive 8 years post the operation, there were no adjuvant patients that survived beyond 5.5 years following the operation (*p* < 0.05) (Fig. 3c).

## Discussion

Protocols combing chemotherapy, radiotherapy and surgical resection became the gold standard in order to obtain an optimal patient outcome. Still, the best regimen remains under debate, due to varying reports on benefits vs toxicity ratios. To this end we have examined the following: the immediate outcomes of pneumonectomy in stage IIIa of NSCLC and the impact of NA therapy on operation complexity; the long-term outcomes of IIIa NSCLC patients undergoing pneumonectomy followed by adjuvant therapy compared to a combined modality treatment of preoperative neoadjuvant treatment followed by pneumonectomy; in efforts to determine the short- and long-term morbidity of such patients and to assess the additive value of neoadjuvant therapy in terms of long term survival.

Our first observation was that NA therapy when added to pneumonectomy does not add complexity to the surgery as was previously reported (evaluated by using both operation length and duration of hospitalization as surrogate markers) [8, 9]. Furthermore, the morbidity in the 1st year after the operation was evaluated by readmissions and transfers between departments, and was found to be not statistically different from the adjuvant group (this might be attributed to the small number of patients in each cohort), however a trend towards increased readmissions and interdepartmental transfer in these admissions was recognized in the NA group. When estimating the peri-OP mortality rate we found it to be equal between the groups, stressing the fact that NA therapy does not increase the peri-op mortality. Nonetheless, when exploring the long term benefits of the NA therapy the survival rate, was found to be 30% eight years post-OP, significantly higher than the adjuvant group that was 0% at 5.5 years post-op [10]. The long-term outcomes of the pneumonectomy + adjuvant treatment group observed in our cohort are in concordance with the historically poor outcomes of single-modality therapy in stage IIIa NSCLC patients [10–12].

Lately, yang et al. have compared right-sided vs left-sided Pneumonectomy after Induction therapy (chemotherapy or chemoradiation) for NSCLC [13]; in a subgroup analysis of stage IIIa patients the right sided pneumectomy had worse 30, 90 days mortality rate compared to left side pneumonectomy but similar 5 year survival, we did not see this phenomenon in our cohort (side difference survival), maybe be due to our cohort size. However, our NA group 30 days mortality was 8.6%, similar to the 8.2% observed in that study and was lower than the one reported in the past as being 38% [14]. It can be explained by the fact that tertiary care has improved significantly over the last twenty years, thus lower morbidity and mortality following complex surgical procedures are detected in experienced high volumes centers[15], moreover patient selection criteria for pneumonectomy have changed as well as pre-operative evaluation, strategies to protect the bronchial stump, and the use of 3-dimensional radiation planning [16].

Surgery is considered most effective when neither N2 or mediastinal disease exist, thus the role of imaging as well as histological analyses are of extreme importance [17–19]. Importantly, in the present study the patients that received chemoradiation, were PET scan verified to no absorbance by structures or lymph nodes in the mediastinum; only then was surgery indicated.

In summary, this single-center experience in advanced stage NSCLC patients demonstrates that operation complexity, morbidity and peri-OP mortality is not affected by NA treatment. Nevertheless, long term survival in the IIIa group receiving NA treatment was significantly longer, as the adjuvant groups had no survivors at 5.5 years post OP and the NA group had 30% survival at 8 years post OP. This might be the result of the synergistic effect of chemoradiotherapy on patient’s fate. When considering earlier reports of this nature, the improved effectiveness of the tri-modality regimen may be the result of the patient cohort (no N2 disease), expert clinical practice and surgical acumen provided at this high-volume medical center as well as the specific NA protocol and timing.

### Study limitations

While this study compared between pneumonectomy patients, with or without prior treatment of chemo-radiotherapy, a multitude of factors can impact patient survival, including surgeon, hospital volume, surgeon experience and postoperative care [15, 20]. In addition, previous multivariate analyses raised male gender, advanced age, low body mass index, American Society of Anesthesiology (ASA) score ≥ 3 NSCLC, extended procedures, associated cardiovascular diseases, bronchial stump reinforcement, absence of systematic lymphadenectomy, and time-period as predictors of 90-day mortality [13, 21]. While the mean age, procedure times and patient gender were similar across cohorts, the other risk factors were not analyzed in the present study, and may have introduced a bias for treatment efficacy in one cohort over the other. Moreover, the study cohort is relatively small and the fact that it is a single institution, single center, single experienced surgeon study might introduce bias to the study; Thus, when implementing this study results one should remember that such operations might be better performed by the more experienced surgeons in the group.

## Conclusion

We conclude that Neoadjuvant chemoradiation therapy followed by pneumonectomy does not increase the surgical complexity, does not increase the peri-OP mortality, but it does increase the long term survival; thus, should be considered as the protocol of choice in stage IIIa NSCLC patients. The importance of this study is by shading some light on the validity of the tri-modality protocol and encouraging using the NA + pneumonectomy regimen when needed.

## Abbreviations

COPD: Chronic obstructive pulmonary disease
FEV1: Forced expiratory volume in 1 s
NA: Neo adjuvant
NSCLC: Non-small cell lung cancer
ppoFEV1: predicted postoperative forced expiratory volume in 1 s
