# Correction to: Trimodally treatment for stage IIIa NSCLC patients increases survival while not effecting surgical mortality or complexity

**DOI:** 10.1186/s13019-019-0856-4

**Published:** 2019-02-12

**Authors:** Dan Aravot, Yaron D. Barac, Efrat Krutzwald-Josefson, Aaron M. Allen, Dov Flex, Nir Peled, Mordechai R. Kramer, Yuri Peysakhovich, Milton Saute

**Affiliations:** 1 Department of Cardiothoracic Surgery, Rabin Medical Center, Beilinson Campus, Petach-Tikva, Israel; 2 Department of Oncology, Rabin Medical Center, Beilinson Campus, Petach-Tikva, Israel; 3 Department of Pulmonary Medicine, Rabin Medical Center, Beilinson Campus, Petach-Tikva, Israel; 4 Sackler Faculty of Medicine, Tel Aviv University, Tel Aviv, Israel

**Correction to: J Cardiothorac Surg (2019) 14:7**

**https://doi.org/10.1186/s13019-018-0829-z**

The original article [1] contained an error whereby all authors’ names were mistakenly inverted. This error has now been corrected.
